# Functional interplay between RND efflux pumps and GacS in *Pseudomonas aeruginosa*

**DOI:** 10.1128/aem.01223-25

**Published:** 2025-08-18

**Authors:** Justyna W. Adamiak, Charles Bergen, Laiba Ajmal, Sidhvi Reddy, Paolla Gruber Anderson, Valentin V. Rybenkov, Helen I. Zgurskaya

**Affiliations:** 1 Department of Chemistry and Biochemistry, University of Oklahoma 522128 , Norman, Oklahoma, USA; Indiana University Bloomington, Bloomington, Indiana, USA

**Keywords:** *Pseudomonas aeruginosa*, multidrug efflux, transcriptional control mechanisms

## Abstract

*Pseudomonas aeruginosa* is an opportunistic human pathogen that is ubiquitous in the environment. The environmental versatility of *P. aeruginosa* is often attributed to a large arsenal of active efflux pumps, low permeability of the outer membrane, and extensive regulatory networks. In this study, we analyzed putative functional interplay between polyspecific drug efflux pumps of the Resistance-Nodulation-Division (RND) superfamily of proteins and the GacSA two-component regulatory system. RND transporters are the major contributors to antibiotic resistance and survival under various environmental stresses, whereas GacSA is a global regulator and governs critical lifestyles during human colonization. We found that the inactivation of either RND pumps or *gacS* induces broad, partially overlapping responses in lifestyle (e.g., rhamnolipid biosynthesis, heat shock response, and quorum sensing), virulence (e.g., proteases, toxins, and phenazines secretion systems), and metabolic programs (e.g., electron, iron capture, synthesis/degradation of ketone bodies, and cyanoamino acid metabolism) that are more pronounced during the exponential than the stationary phase. GacSA and RND efflux pumps induced opposite responses in the gene expression of *P. aeruginosa*, whereas the overexpression of GacS was additive with the deletion of efflux pumps in gene expression and under growth conditions typically associated with human infections such as elevated temperature and iron deprivation. We conclude that RND efflux and GacSA networks partially overlap with each other.

**IMPORTANCE:**

RND efflux pumps are the major contributors to intrinsic and clinical levels of antibiotic resistance of *P. aeruginosa,* and their expression is regulated by various environmental factors. However, functional relationships between active efflux pumps and regulatory networks involved in virulence and pathogenesis of *P. aeruginosa* remain unclear. This study showed that the loss of RND-dependent efflux functions or the inactivation of the *gacS* pathway induces partially overlapping transcriptional and physiological responses, suggesting that these pathways could be complementary to each other in hostile environments.

## INTRODUCTION

*Pseudomonas aeruginosa* is an opportunistic pathogen that causes nosocomial infections and produces chronic infections in hospitals and in cystic fibrosis (CF) patients. Due to its impact on human health, this clinical pathogen is listed among the “high-priority” group of pathogens by WHO, which requires immediate attention and the development of novel antibiotics in clinics (1). Unfortunately, the selection of the most appropriate antibiotic is complicated because of the ability of *P. aeruginosa* to develop resistance to multiple classes of antibacterial agents. The low-permeability outer membrane (OM) and an arsenal of drug transporters, especially from the Resistance Nodulation Division (RND) superfamily, are the major contributors to intrinsic and acquired levels of antibiotic resistance, whereas target mutations and enzymatic modifications further enable clinical resistance to specific antibiotic classes (2–4).

To date, 12 RND-efflux pumps have been best characterized, out of which 11 pumps are capable of multidrug efflux (5). Some of these pumps are constitutively expressed, others are inducible by certain stress conditions, whereas certain pumps are overproduced due to mutations in regulatory proteins (6–8). There is emerging evidence that certain RND efflux pumps are also involved in the efflux of bacterial factors important for the virulence and pathogenicity of the bacterium (6, 9). However, how and to what extent active efflux contributes to the establishment and progression of infections remains unclear.

*P. aeruginosa* encodes an extensive array of two-component signaling systems (TCS), more than any other known bacterial pathogen (10–12). These TCS play crucial roles in virulence and antibiotic resistance, regulating the transition between acute and chronic infection states. Among these, the GacSA system is a key regulatory system that translates environmental cues into specific cellular responses (13, 14). GacS, a sensor kinase, activates the response regulator GacA, which is involved in the regulation of the metabolic switch between primary and secondary metabolism, and contributes to *P. aeruginosa* virulence, quorum sensing (QS), and biofilm formation. Loss of GacS results in diminished virulence and ecological fitness. Recently, it has also been reported that *gacS* is among a few genes frequently mutated in the cystic fibrosis clinical isolates and is one of the hot spots of mutations during chronic CF lung infections (15).

Individually maintained laboratory sublines of PAO1 undergo microevolution during cultivation and genetic manipulation, leading to selective mutations that cause genetic and phenotypic variations (16, 17). Such adaptations are essential for survival during infections and in external environments. Given the significance of these adaptations, particularly the frequent mutations in the *gacS* gene in CF contexts (15), we investigated genetic differences between PAO1-89 and a derived efflux-deficient strain, P1116 (Δ*mexAB-oprM ΔmexXY ΔmexCD-oprJ ΔmexEF-oprN ΔmexJK ΔtriABC*) (18), for both desired and undesired mutations. Sequencing of PAO1 and P1116 genomes performed in this study revealed a frameshift single base pair deletion in the *gacS* gene, inactivating this regulatory system in both strains. This inactivation likely has significant implications for the virulence and adaptability of *P. aeruginosa*.

The aim of this study was to investigate a putative functional link between the RND efflux pumps and the GacSA regulatory system and to establish whether the frameshift mutation in *gacS* could contribute to the phenotypes of P1116. We found that both mutations, the inactivation of RND pumps and *gacS*, induce broad, partially overlapping responses in lifestyle, virulence, and metabolic programs that are more pronounced during the exponential than the stationary phase. Notably, mutations in *gacS* and RND efflux pumps induced opposite responses in the gene expression of *P. aeruginosa*, whereas the overexpression of GacS was additive with the deletion of efflux pumps. In contrast, the physiological behavior of the mutants under stress conditions typically associated with the human host environment, such as osmotic and acidic shock, elevated temperature, and iron deficiency, was largely independent of each other.

## RESULTS

### The expression of *gacS* has a modest effect on the growth phenotype but does not affect the antibiotic susceptibility of *P. aeruginosa*

During the whole genome sequencing of laboratory *P. aeruginosa* strains, we found that the PAO1-89 strain (thereafter called *gacS*^fs^) (19) and its derivative P1116 lacking six major RND efflux pumps (Δ*mexAB-oprM ΔmexXY ΔmexCD-oprJ ΔmexEF-oprN ΔmexJK ΔtriABC*) (thereafter called Δ6*gacS*^fs^) (18) (Table 1) contained one frame-shift deletion (*gacS* 1570del) leading to the deactivation of the *gacS* gene (Table 2). In addition, we also detected single-nucleotide substitutions in eight other genes of Δ6*gacS*^fs^. Two of the substitutions in the *mexA* and *mexL* genes encoding the periplasmic membrane fusion protein of MexAB-OprM efflux pump and the transcriptional regulator of MexEF-OprN efflux pump, respectively, were unlikely to contribute to the Δ6*gacS*^fs^ phenotype because these efflux pumps have been deleted in this strain. Other substitutions may or may not affect the functions of the respective genes. However, a frame-shift deletion in the *gacS* gene indicated that this regulatory system is inactive in Δ6*gacS*^fs^.

**TABLE 1:** List of *P. aeruginosa* strains and plasmids used in this study

Strain or plasmid	Description	Source
*gacS* ^+^	Wild-type PAO1-96	(19)
*gacS^fs^*	PAO1-89 *gacS*1570del	(19)
Δ6*gacS^fs^*	PAO1116 Δ*mexAB-oprM* Δ*mexCD-oprJ* Δ*mexEF-oprN* Δ*mexJKL* Δ*mexXY* Δ*triABC gacS*1570del	(18)
Δ*gacS*	PAO1-89 Δ*gacS*	This study
Δ6Δ*gacS*	PAO1116 Δ*gacS*	This study
*gacS^fs^::*LAC*-gacS*	PAO1-89 *att*Tn7::mini-Tn7T-Gm^r^-LAC-*gacS*	This study
Δ6*gacS^fs^::*LAC*-gacS*	PAO1116 *att*Tn7::mini-Tn7T-Gm^r^-LAC-*gacS*	This study
pUC18T-mini-Tn*7*T-LAC	Ap^r^ Gm^r^ mini-Tn*7*T suicide delivery vector	(20)
pTNS3	Helper plasmid containing Tn*7* transposase complex	(21)
pEXG2	Gm^r^ Allele exchange vector sucrose counterselection	(22)
pUC18T-mini-Tn*7*T-LAC-*gacS*	pUC18T mini-Tn7T- LAC- Gm^r^-vector carrying *gacS* gene	This study
pEXΔ*gacS*	pEXG2 with the upstream and the downstream fragments of *gacS*	This study

**TABLE 2:** List of unique nonsynonymous genetic modifications in *P. aeruginosa* Δ6*gacS^fs^* strain

Reference position	Variant	Locus	Gene/encoded product	Nucleotide change	Amino acid change
472364	SNV	PA0425	*mexA*; multidrug resistant protein MexA^*a*^	A>G	Gln>Arg
1014183	Deletion	PA0928	*gacS*; sensor/response regulator hybrid	del	Ala>fs
1733418	SNV	PA1590	*braB*; branched chain amino acid transporter	A>G	Thr>Ala
1852838	SNV	PA1706	*pcrV*; type III secretion protein PcrV	C>T	Pro>Leu
2857159	SNV	PA2530	Hypothetical protein	G>C	Arg>Pro
2857162	SNV	PA2530	Hypothetical protein	A>C	Asp>Ala
3583282	SNV	PA3191	*gtrS*; glucose transport sensor, GtrS	T>C	Val>Ala
4120608	SNV	PA3678	*mexL*; repressor of the TetR family MexL^*b*^	C>A	Ala>Asp
5159692	SNV	PA4601	*morA*; motility regulator	G>A	Ala>Thr

^*a*^ Most of *mexA* is deleted in Δ6*gacS*^fs^.

^*b*^ *mexEF-OprN* pump, which is regulated by *mexL*, is deleted in Δ6*gacS*^fs^ (23).

**Fig 1:**
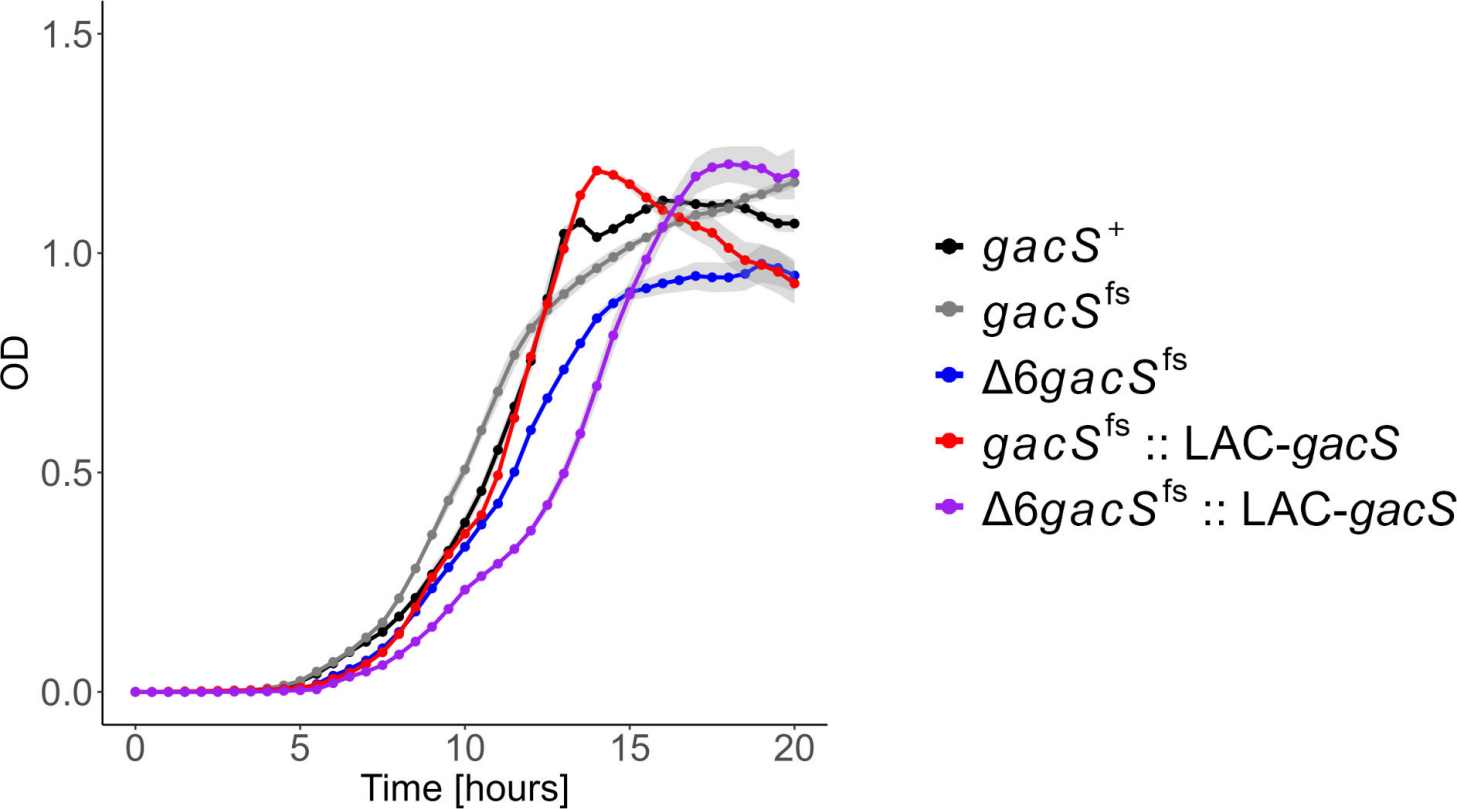
Growth curves of *gacS* mutants in LB media. Cells were induced with 0.1 mM isopropyl β-D-1 thiogalactopyranoside (IPTG).

To complement the *gacS*^fs^ mutation, we cloned the functional *gacS* from PAO1 strain (*gacS*^+^) under an IPTG-inducible LAC promoter and integrated the Plac-*gacS* construct onto the Tn7 sites of *gacS^fs^* and Δ6*gacS^fs^* chromosomes, generating *gacS^fs^::LAC-gacS* and Δ6*gacS^fs^::LAC-gacS* strains, respectively. As revealed by RNA-seq, the Δ6*gacS^fs^::LAC-gacS* cells grown in the presence of 0.1 mM IPTG contained ~4-fold higher levels of *gacS* transcripts than *gacS^+^* (Fig. S1). We also deleted the entire *gacS* gene from PAO1-89 and P1116, nicknamed Δ*gacS* and Δ6Δ*gacS*, respectively. We found that all *P. aeruginosa* strains had similar growth rates when grown in LB broth at 37°C (Fig. 1; Fig. S2). However, *gacS^fs^* and Δ*gacS* strains had somewhat faster growth rates than *gacS*^+^ and the complemented *gacS^fs^::LAC-gacS* strains, suggesting that this mutation might be positively selected under these conditions. In contrast, the growth rates of Δ6*gacS^fs^* and Δ6Δ*gacS* were identical, slower than those of efflux-proficient variants, and reached the stationary phase at lower optical densities. In contrast, the complemented Δ6*gacS^fs^::LAC-gacS* strain had the slowest growth rate. Thus, a frame-shift mutation and the whole gene deletion of *gacS* yield the same growth phenotypes, whereas the effect of the overproduction of GacS is strain-specific and has a notable negative growth effect in the absence of efflux. For all strains, we also analyzed cell morphology and found it preserved (Fig. S3).

The parental, complemented, and deletion *P. aeruginosa* strains were next analyzed for their susceptibilities to various antibiotics, the substrates of RND efflux pumps (Table 3). We found that MICs of novobiocin, ciprofloxacin, chloramphenicol, and azithromycin were identical in *gacS*^+^ and *gacS*^fs^ strains. As expected, Δ6*gacS*^fs^ cells were hypersusceptible to all four antibiotics by orders of magnitude (Table 3). The overproduction of GacS did not significantly affect the MICs of antibiotics in *gacS^fs^::LAC-gacS* and Δ6*gacS^fs^::LAC-gacS* cells. The differences in MICs are at most 2-fold, which is within the error of the 2-fold dilution method used to determine the MICs. Similarly, the full deletion of *gacS* in Δ*gacS* and Δ6Δ*gacS* cells had no effect on the MICs of antibiotics. Thus, genetic inactivation or overproduction of GacS does not lead to changes in antibiotic susceptibility of *P. aeruginosa*.

**TABLE 3:** Minimal inhibitory concentrations (μg/mL) of antibiotics against PAO1 and indicated efflux-deficient strains

Genotype	MIC of^*a*^:			
	NOV	CIP	CHF	AZI
*gacS* ^+^	>1,024	0.125	25	256
*gacS^fs^*	>1,024	0.125	25	256
Δ*gacS*	>1,024	0.125	12.5–25	256
*gacS^fs^::*LAC*-gacS*	>1,024	0.125	25	256
Δ6*gacS^fs^*	32–64	0.004	0.8–1.6	2
Δ6Δ*gacS*	32	0.008–0.016	0.8	4
Δ6*gacS^fs^::*LAC*-gacS*	32	0.008	1.6	4–8

^*a*^ NOV, novobiocin; CIP, ciprofloxacin; CHF, chloramphenicol; AZI, azithromycin.

### Inactivation of RND efflux pumps and *gacS* inactivation are orthogonal to the onset of the stationary phase

**Fig 2:**
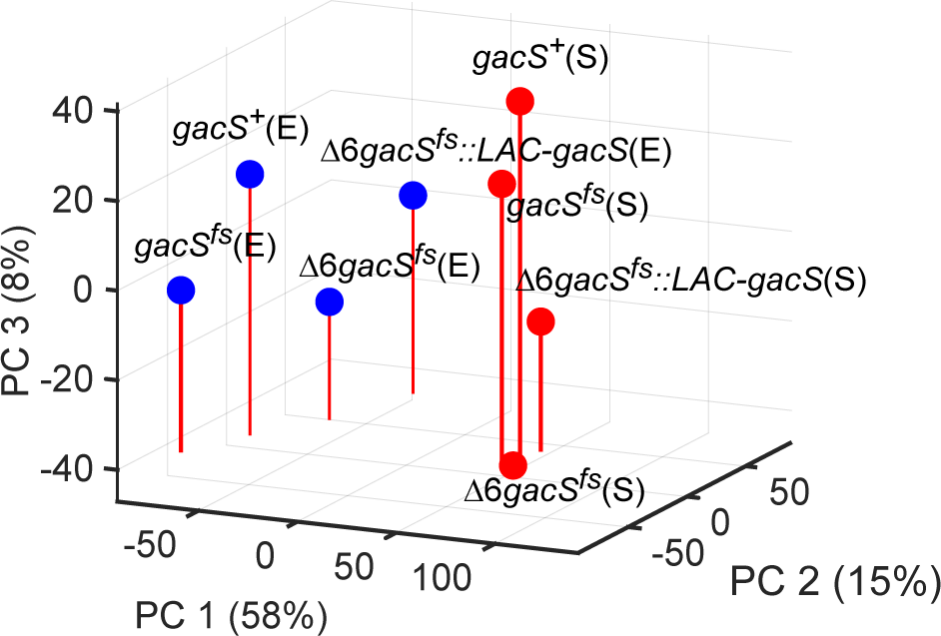
Principal component analysis of gene expression in *gacS* and efflux pump mutants. Exponential and stationary phase samples are shown as blue and red symbols, respectively.

We next analyzed the transcriptional profiles of four strains in both exponential and stationary growth phases: *gacS*^+^, *gacS^fs^*, ∆6*gacS^fs^*, and Δ6*gacS^fs^::LAC-gacS*, using *gacS*^+^ as a reference. The analysis of the overall transcriptional profiles indicated that gene expression differences between strains were much greater for the exponential than the stationary phase cells, whereas the transition to the stationary phase dominated transcriptional responses caused by mutations in efflux or *gacS*. Principal component analysis (PCA) of the log_2_-averaged normalized counts in all eight RNA samples highlighted this difference related to growth states (Fig. 2). Principal component (PC) 1 explained 58% of the total variance, where samples were grouped according to their growth state. The stationary phase samples grouped tightly with each other, and the exponential samples dispersed primarily along PC 2 (15% of the total variance), which was related to the effect of *gacS* and ∆6 mutations. The *gacS^fs^* and Δ6*gacS^fs^::LAC-gacS* strains for both exponential and stationary phases were located on opposite extremes along PC 2, suggesting opposing influences on *P. aeruginosa* physiology. The changes along PC 3 (8%) were collinear in *gacS* and ∆6 mutants in the stationary phase but occurred only in the *gacS* mutants in the exponential phase.

### Transcriptional response of the efflux and *gacS* mutants

**Fig 3:**
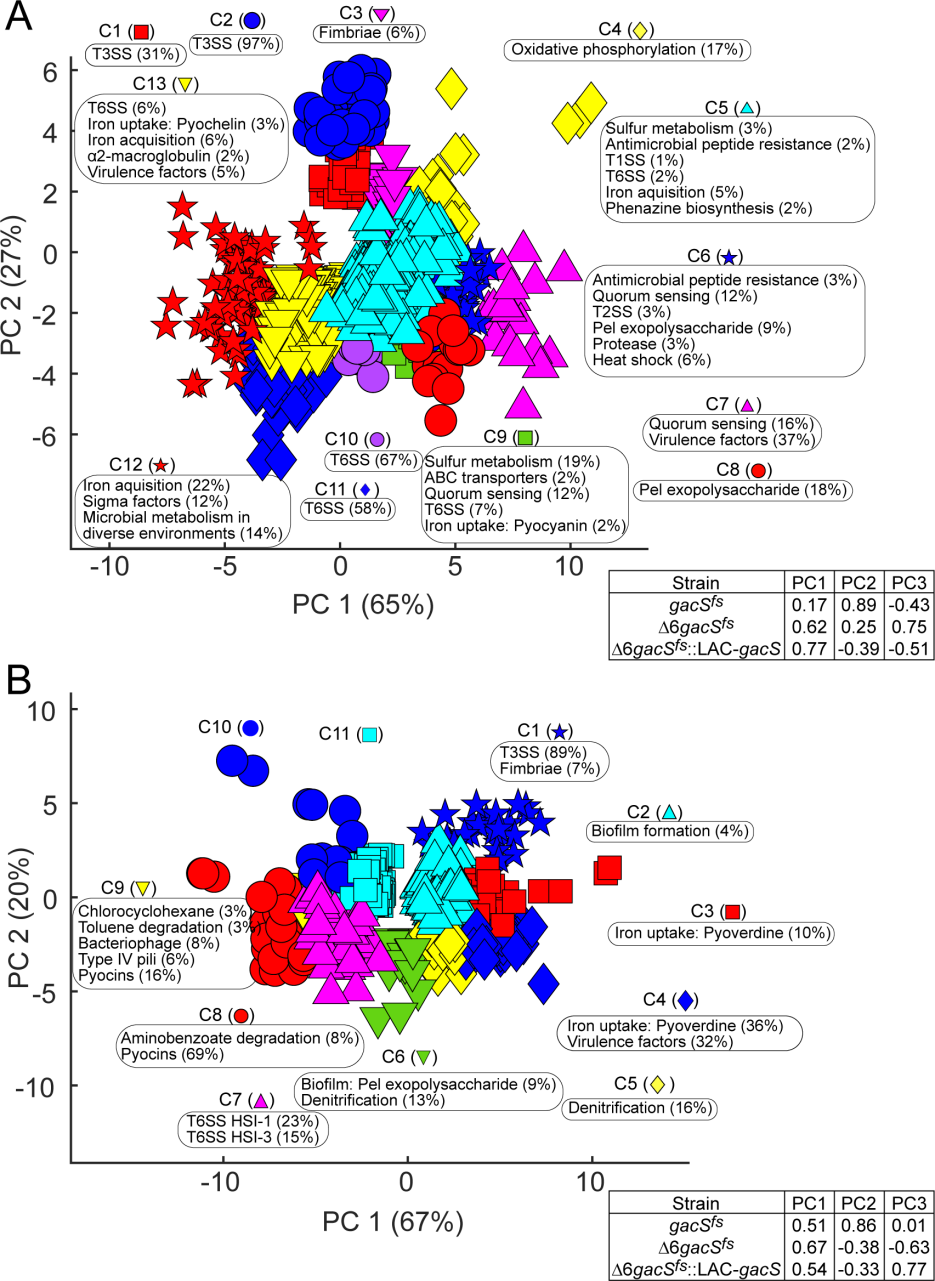
Hierarchical clustering of differentially expressed genes in exponential phase (**A**) or stationary phase (**B**) samples. Clusters are listed with enriched pathways along with their representation expressed as the percent of genes within the cluster.

Using a combination of pairwise differential expression and clustering analyses (24), we identified 842 and 589 genes, in exponential and stationary phases, respectively, that were significantly affected (>2-fold, FDR < 0.05) by at least one of the mutations and clustered apart from the unaffected genes (see Materials and Methods). We identified 13 and 11 clusters, respectively, in the exponential (Fig. 3A) and stationary (Fig. 3B) phase samples. The largest subset of the genes, 599, was upregulated during the exponential phase, compared with 369 genes that were downregulated (Fig. S4). The three mutants showed a substantial, >50%, overlap among affected genes, with the greatest similarity observed between upregulated genes in exponential ∆6*gacS^fs^* and Δ6*gacS^fs^::LAC-gacS* cells and downregulated genes in exponential *gacS^fs^* and ∆6*gacS^fs^* cells (Fig. S4). Thus, mutations in *gacS* and efflux transporters affect similar genetic programs.

We did not find a notable effect of *gacS* and/or efflux pump mutations on the cell envelope stress responses. In *P. aeruginosa,* AlgU, SigX, and SbrI are extracytoplasmic sigma factors that play the major role in establishing a cell envelope stress response (25). Although the *algU* transcript was slightly elevated in the exponential *gacS^fs^* cells, all three sigma factors and their respective regulons were either unchanged or notably repressed in exponential and stationary ∆6*gacS^fs^* and Δ6*gacS^fs^::LAC-gacS* (Table S1), suggesting that inactivation of efflux does not generate and even suppresses a cell envelope stress response.

### Exponential phase

Pathway enrichment analysis was next performed on each of the 13 clusters for exponential samples, yielding 42 enriched pathways (Fig. 3A). Virulence-related pathways accounted for the majority of significantly differentially expressed genes in all three mutant strains, followed by metabolism and lifestyle. Pathways from all three affected categories often co-clustered with each other, suggesting potential coregulation of these genes.

**Fig 4:**
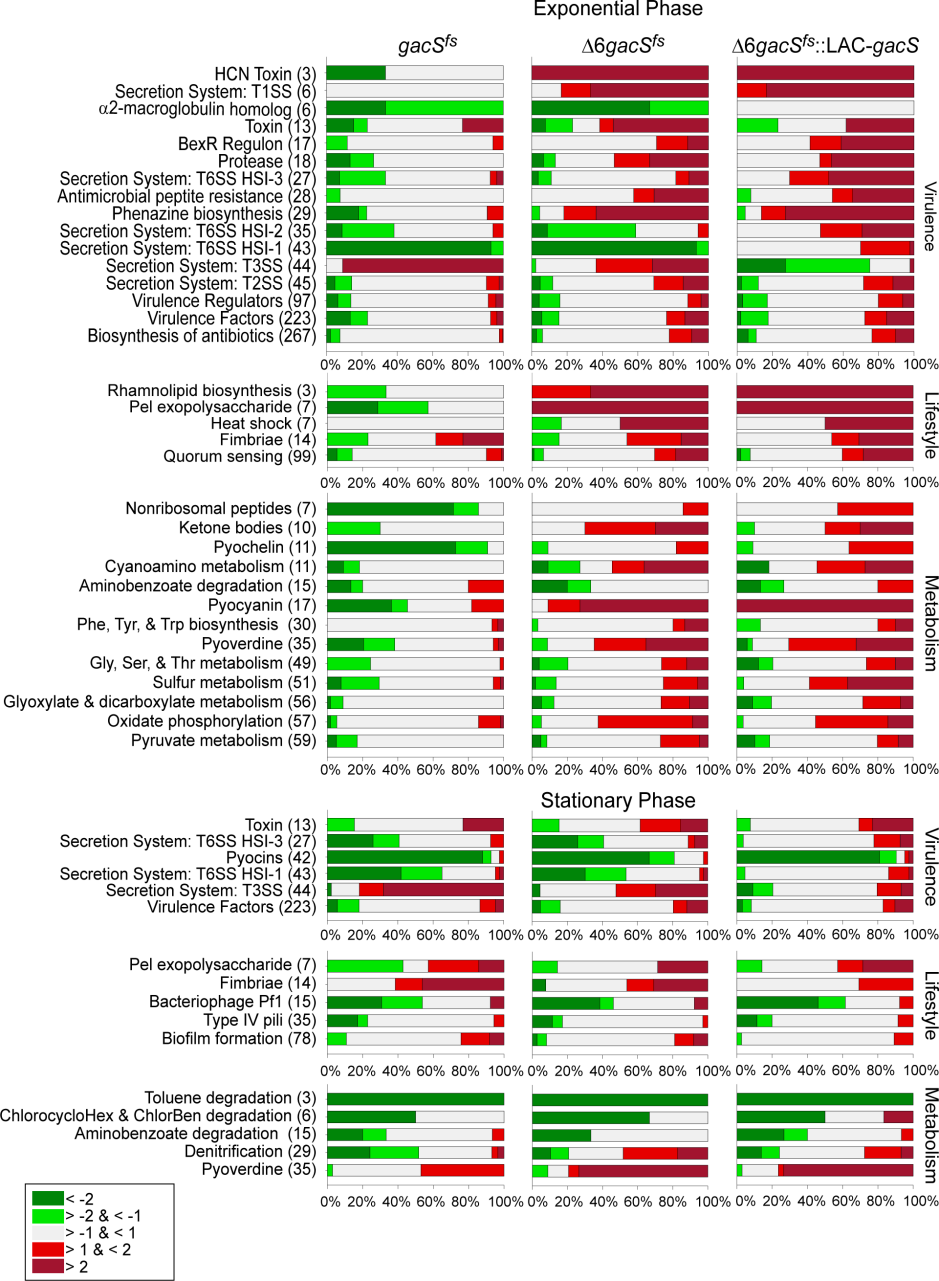
Expression profiles of enriched pathways in exponential or stationary phase samples. Pathways are listed with their corresponding number of genes. Up- or down-regulation is specified by color. Values on the x axis indicate the percent of genes in a regulon.

Virulence factors such as proteases, toxins, and phenazines exhibited upregulation in both ∆6*gacS*^fs^ and Δ6*gacS^fs^::LAC-gacS* mutants (Fig. 4, top). There were distinct patterns in the expression of Type VI Secretion Systems (T6SS). Specifically, T6SS HSI 1 and 2 were primarily downregulated in *gacS*^fs^ and ∆6*gacS*^fs^, whereas T6SS HSI 1, 2, and 3 were upregulated in Δ6*gacS^fs^::LAC-gacS*. In contrast to T6SS, the type III secretion system (T3SS) showed different expression profiles, with upregulation in both *gacS*^fs^ and ∆6*gacS*^fs^ mutants, whereas it was downregulated in Δ6*gacS^fs^::LAC-gacS*. Overall, the loss of *gacS* in *P. aeruginosa* was associated with a decrease in virulence, and the ∆6 mutations were linked with an increased virulence response.

Lifestyle-related pathways, such as rhamnolipid biosynthesis, heat shock response, fimbriae, and quorum sensing, displayed upregulation in both ∆6*gacS*^fs^ and Δ6*gacS^fs^::LAC-gacS* mutants. The *pel* exopolysaccharide pathway, crucial for biofilm stability and production, showed downregulation in the *gacS*^fs^ mutant but upregulation in the Δ6*gacS^fs^::LAC-gacS* mutant. Although the loss of *gacS* had a minimal impact on lifestyle pathways, the ∆6 mutations induced significant upregulation across all of them (Fig. 4, top).

Metabolic pathways exhibited primarily upregulated expression in both ∆6*gacS*^fs^ and Δ6*gacS^fs^::LAC-gacS* mutants. Notably, pathways associated with electron, iron (pyocyanin and pyoverdine), and sulfur capture showed the most significant upregulation. Additionally, synthesis and degradation of ketone bodies and cyanoamino acid metabolism were among the pathways upregulated in ∆6*gacS*^fs^ and Δ6*gacS^fs^::LAC-gacS* mutants. In contrast, in the *gacS*^fs^ mutant sample, most of the metabolic pathways were predominantly downregulated.

To summarize, the exponential phase samples revealed distinct and opposite effects of ∆6 and *gacS* inactivation (Fig. 4, top, and Fig. 6). The ∆6 phenotype was associated with an increased expression of lifestyle, virulence, and metabolic pathways. Conversely, *gacS* inactivation was linked to decreased expression of virulence and metabolic pathways, with little or no change in lifestyle pathways. T3SS, T6SS, and macroglobulin expression showed similar expression patterns in *gacS*^fs^ and ∆6*gacS*^fs^, suggesting that they are primarily controlled by GacS. The rest of the pathways showed greater similarity in ∆6*gacS*^fs^ and ∆6*gacS*^fs^::LAC*-gacS*, revealing the primary regulatory role of efflux inactivation.

### Stationary phase

Transcriptional analysis of stationary phase samples revealed fewer significantly enriched genes (589), resulting in 11 clusters (Fig. 3B). Pathway enrichment analysis of all 11 stationary phase clusters identified 16 enriched pathways related to virulence, lifestyle, and metabolism. Enriched pathways within clusters 1–5 were mostly upregulated, with a few pathways displaying mixed expression results. Conversely, clusters 7–9 were predominantly characterized by downregulated pathways, with most of them being associated with metabolism. Pathway enrichment analysis did not identify any enriched pathways for clusters 10 and 11.

In the stationary phase, we observed a more diverse pattern of gene expression changes that did not exhibit a uniform trend in most pathways (Fig. 4, bottom). Additionally, fewer pathways showed significant enrichment compared with samples in the exponential phase. Notably, T3SS and T6SS virulence pathways again displayed a similar regulation in *gacS*^fs^ and ∆6*gacS*^fs^ cells, which differed from Δ6*gacS^fs^::LAC-gacS*.

Regarding lifestyle-related pathways, all three strains exhibited similar expression results. Bacteriophage Pf1 and type IV pili were mostly downregulated in strains, with biofilm pathways showing mixed expression responses. Fimbriae had the greatest upregulation in *gacS*^fs^, with it slightly decreasing in ∆6*gacS*^fs^ and more so in Δ6*gacS^fs^::LAC-gacS*. Finally, all three strains had similar expression results for metabolic pathways. Toluene, chlorocyclohexane, and aminobenzoate degradation were all significantly downregulated, whereas denitrification was mixed. Pyoverdine, however, was significantly upregulated in all three strains.

In the stationary phase (Fig. 4, bottom, and Fig. 6), fewer pathways were significantly enriched compared with samples in the exponential phase, and the results demonstrated mixed expression responses. The *gacS* inactivation was associated with broad downregulation across multiple pathways, with only several pathways, including fimbriae and exopolysaccharide biosynthesis, production of pyoverdine and toxins, and T3SS, showing a notable upregulation. Notably, the deletion of efflux pumps showed a similar effect on gene expression. T3SS was the only exception to this pattern and did not appear to be induced in the Δ6 strain.

### Mutations in *gacS* and efflux pumps alter the secretion of secondary metabolites and proteases

Our transcriptomics results are consistent with previous studies that implicated GacS in the activation of various virulence factors as well as chronic persistence genes (26, 27). We next analyzed how *gacS* inactivation affects the production of pyocyanin, one of the major virulence factors of *P. aeruginosa,* regulated by GacSA. Our results show that the inactivation of *gacS* in the *gacS*^fs^ strain led to a significant decrease in the production of pyocyanin compared with the *gacS*^+^ cells (Table 4). The inactivation of efflux in Δ6*gacS*^fs^ partially counteracted the effect of the GacS loss on pyocyanin production, as seen from higher levels of this pigment produced by Δ6*gacS*^fs^ cells. This result agrees with our transcriptomics findings that showed distinct and opposite effects of ∆6 and *gacS* inactivation on gene expression in growing cells. The specificity of the reduced pyocyanin phenotype to g*acS^fs^* was further validated by measuring pyocyanin levels in the complemented strains. Overproduction of GacS in either *gacS^fs^::LAC-gacS* or Δ6*gacS^fs^::LAC-gacS* restored the pyocyanin secretion to the *gacS^+^* levels.

**TABLE 4:** Growth physiology of *P. aeruginosa* strains and secretion of metabolites and proteases

Phenotype or a stress factor^*a*^	Time of growth/ concentration of stressor	*gacS* ^+^	*gacS* ^fs^	Δ6*gacS^fs^*	*gacS^fs^* :: LAC-*gacS*	Δ6*gacS^fs^* :: LAC-*gacS*
Pyocyanin production^*b*^	24 h	0.23 ± 0.03	0.05 ± 0.01	0.16 ± 0.02	0.14 ± 0.02	0.23 ± 0.05
Pyoverdine production^*c*^	24 h	0.12 ± 0.01	0.13 ± 0.03	0.23 ± 0.02	0.11 ± 0.01	0.15 ± 0.00
	48 h	0.40 ± 0.1	0.15 ± 0.04	0.61 ± 0.11	0.22 ± 0.03	0.25 ± 0.04
Pyochelin production^*d*^	24 h	0.30 ± 0.02	0.3 ± 0.05	0.51 ± 0.06	0.39 ± 0.00	0.56 ± 0.00
	48 h	0.43 ± 0.07	0.38 ± 0.04	0.9 ± 0.15	0.32 ± 0.06	0.59 ± 0.15
Protease^*e*^ (U/ml)	24 h	0.84 ± 0.02	0.43 ± 0.01	0.95 ± 0.11	0.70 ± 0.00	0.94 ± 0.02
LB broth, 37°C	24 h	10.45	10.67	8.60	10.09	8.51
Biofilm formation^*f*^	48 h	1.06 ± 0.25	1.19 ± 0.21	0.56 ± 0.05	0.86 ± 0.30	0.72 ± 0.05
Bile salts	24 h/0.125%	8.08	7.54	3.61	6.88	3.36
	24 h/0.5%	3.76	3.55	1.27	5.00	1.25
2,2’-dipyridyl	24 h/125 µM	9.63	9.93	3.52	8.53	3.93
	24 h/250 µM	9.06	8.56	0.40	7.86	0.86
pH 4.6	24 h	8.92	7.38	4.50	6.98	3.61
0.5 M NaCl	24 h	9.68	7.86	7.79	8.76	7.72
41°C	24 h	10.56	11.65	7.77	9.49	8.65

^*a*^ Area under the curve (AUC) values for the growth curves of the analyzed strains in the presence of 0.1 mM IPTG. Conditions as in Avican et al. (28).

^*b*^ Production of pyocyanin in LB broth, 37ºC. The quantity of pyocyanin was measured at 520 nm and normalized to cell density registered at 600 nm. Results shown in the table are expressed as OD_520_/OD_600_ values.

^*c*^ The quantity of pyoverdine was determined by measuring the absorbance at 405 nm and normalizing to cell density measured at 600 nm. Results shown in the table are OD_405_/OD_600_ ratios.

^*d*^ The quantity of pyochelin was measured at OD 313 nm and normalized to cell density measured at 600 nm. Results shown in the table are OD_313_/OD_600_ ratios.

^*e*^ Protease activity was determined using the azocasein assay and by measuring the absorbance at 430 nm. One unit of protease was defined as the enzyme quantity that produces an increase of 1 OD unit per hour.

^*f*^ Biofilm formation was tested using the Calgary Biofilm Device. Numbers are OD units at 590 nm.

Several studies have suggested that both GacS and the RND efflux pumps play a role in iron acquisition by *P. aeruginosa* (23, 29, 30). Measurements of concentrations of two siderophores, pyochelin and pyoverdine, in *gacS* and efflux strains showed that the effect of *gacS* is dependent on the activity of efflux pumps (Table 4). The levels of secretion of both siderophores were very similar in *gacS^+^* and *gacS*^fs^ cultures but notably increased in the efflux-deficient Δ6*gacS^fs^*. The levels of the siderophores in the complemented strains depended on the genetic background. Although the amounts of secreted siderophores in *gacS^fs^::LAC-gacS* were similar to those in *gacS^+^* and *gacS*^fs^ cultures, the overproduction of GacS in Δ6*gacS^fs^::LAC-gacS* brought the levels of secretion down to those of efflux-proficient cells. This result suggests that the activation of GacSA could alleviate the iron acquisition response triggered by efflux inactivation.

Analyses of protease secretion showed that inactivation of *gacS* reduces the production of this virulence factor in *gacS*^fs^ cells, whereas the overproduction of GacS in *gacS^fs^::LAC-gacS* cells at least partially restored the protease amounts (Table 4). However, the spent cultures of Δ6*gacS^fs^,* Δ6*gacS^fs^::LAC-gacS,* and *gacS^+^* cells contained similar protease amounts, suggesting that efflux inactivation dominates in the regulation of the protease secretion and that the contribution of GacS to the regulation of protease production requires activities of efflux pumps.

### GacS does not contribute to survival during infection-related stresses

The role of active efflux in virulence has been demonstrated in various gram-negative pathogens, including *P. aeruginosa* (27, 31–33). The inactivation of multiple RND pumps also leads to growth deficiencies under infection-related stresses (34). We next analyzed whether the functions of RND efflux pumps and GacS pathway overlapped in responses to infection-associated stresses. For this purpose, we measured biofilm formation and collected growth curves of all strains under five stress conditions (Table 4): (i) bile salts (0.5% and 0.125%); (ii) low iron (125 µM and 250 µM 2,2’-dipyridyl); (iii) acidic stress (pH 4.6); (iv) osmotic stress (0.5 M NaCl); and (v) high temperature (41°C). We found that Δ6*gacS^fs^* cells produce the lowest amounts of biofilms (~40% reduction), and this deficiency was partially complemented in the complemented Δ6*gacS^fs^::LAC-gacS* cells. However, no significant differences in biofilm production were seen between *gacS*^+^, *gacS*^fs^, and complemented strains. This result is consistent with our transcriptomics data that showed no significant differences between the strains in the genes associated with biofilm production (Fig. 4, bottom).

**Fig 5:**
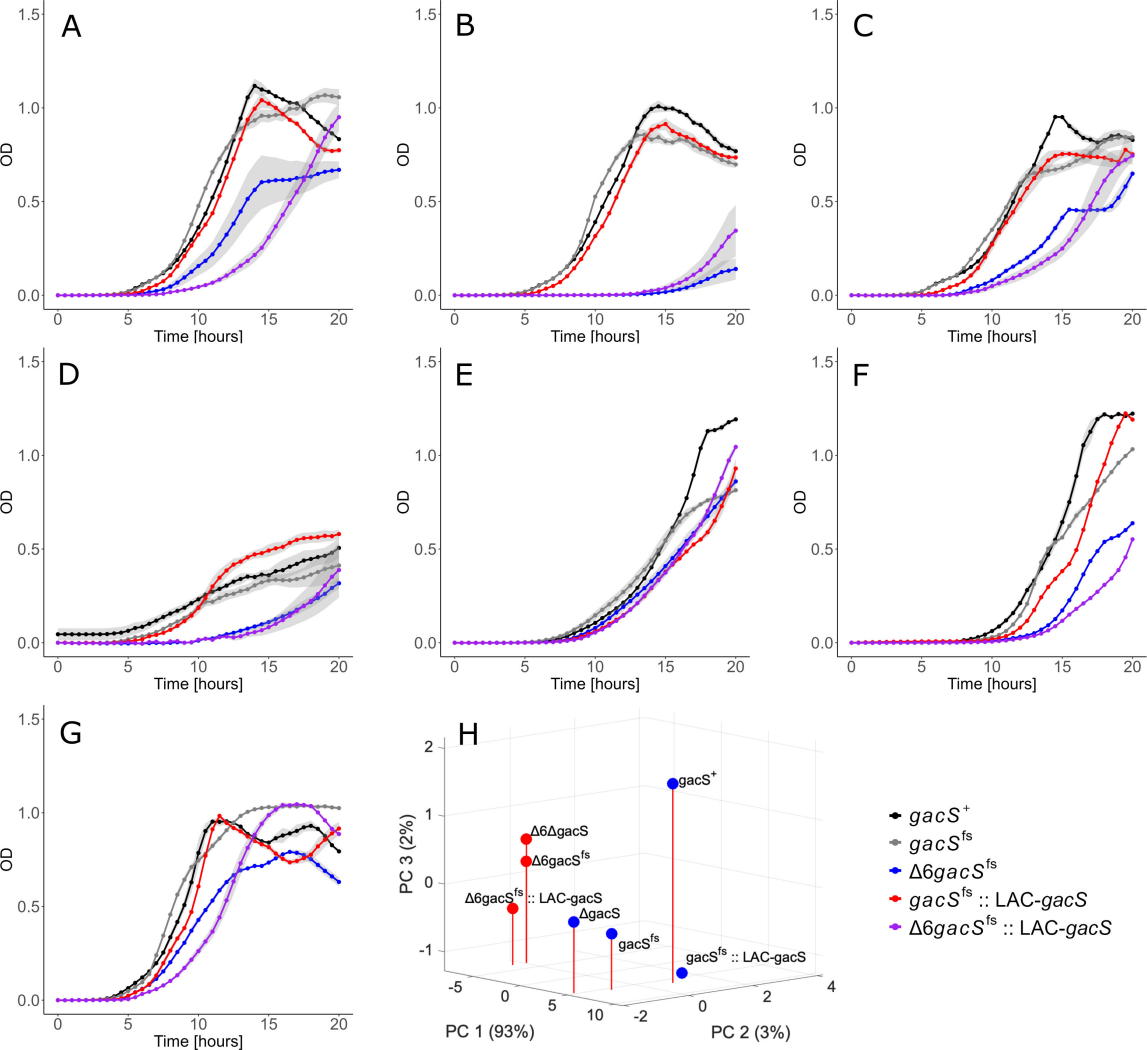
Growth physiology of *P. aeruginosa gasS* and Δ6 strains. (**A and B**) Limited iron conditions in the presence of 125 µM and 250 µM iron chelator 2,2’-dipyridyl, respectively. (**C and D**) Bile salt stress in the presence of 0.125% and 0.5% sodium deoxycholate. (**E**) Osmotic stress in the presence of 0.5 M NaCl. (**F**) Acidic stress in LB broth pH = 4.6. (**G**) High temperature stress in LB broth 41°C. Overnight cultures were diluted to an OD_600_ of 0.001 and inoculated at 5 × 10^3^ cells into a 96-well plate containing LB broth with indicated stress conditions. Optical densities were monitored in a Tecan Spark 10M plate reader at 37 ˚C every 30 min. Error bars are SD (*n* = 3). If necessary, cells were induced with 0.1 mM isopropyl β-D-1 thiogalactopyranoside (IPTG). Error bars represent the standard error of at least two biological replicates, each with at least two technical replicates. (**H**) Principal component analysis of the area under the curve (AUC) of strains under different stress conditions.

For planktonic cultures, no growth deficiencies were seen under conditions of osmotic stress, suggesting that neither RND efflux nor the GacS pathway is required under these conditions (Fig. 5; Fig. S2). In agreement with previous studies (23), the three RND-deficient strains Δ6*gacS*^fs^ and its Δ6Δ*gacS* and Δ6*gacS^fs^::LAC-gacS* derivatives showed notable growth deficiencies when compared with the respective efflux-proficient variants under all other stress conditions. The largest growth defects of efflux-deficient cells can be seen under iron deficiency and bile salt conditions, followed by acidic stress and high temperature. We found no gross changes in cell morphology under these stress conditions (Fig. S3). However, cell enlargement was seen for *P. aeruginosa* exposed to high concentrations of bile salts and 0.5 M NaCl.

The overexpression of *gacS* in Δ6*gacS^fs^::LAC-gacS* cells partially alleviated the efflux-dependent growth defects in the presence of 250 µM 2,2’-dipyridyl (Fig. 5A; Table 4), but the complementation effect was modest and delayed at the lower 125 µM 2,2’-dipyridyl concentration. In addition, the overexpression of *gacS* slightly slowed the growth of *P. aeruginosa* at high temperature and acidic stress (Fig. 5F and G), but this negative effect is seen in both efflux plus and minus backgrounds. Thus, in agreement with RNA-seq data, efflux pumps are essential for metabolic activities of *P. aeruginosa* and are opposite to the expression of *gacS*. In contrast, *gacS*^+^, *gacS*^fs^, Δ*gacS,* and *gacS^fs^::LAC-gacS* did not have notable differences in growth rates under the stresses. However, in most cases, *gacS*^+^ and *gacS^fs^::LAC-gacS* cells had the highest cell densities during and after transition into the stationary phase.

To identify possible hidden features, for all growth curves, we calculated the area under the curve values (AUC) and used these values in PCA (Fig. 5H). We found that all efflux-deficient strains clustered close to each other, whereas the efflux plus strains showed a broader spread. PC1 (93%) strongly dominated the clustering and separated efflux-proficient and -deficient strains.

## DISCUSSION

**Fig 6:**
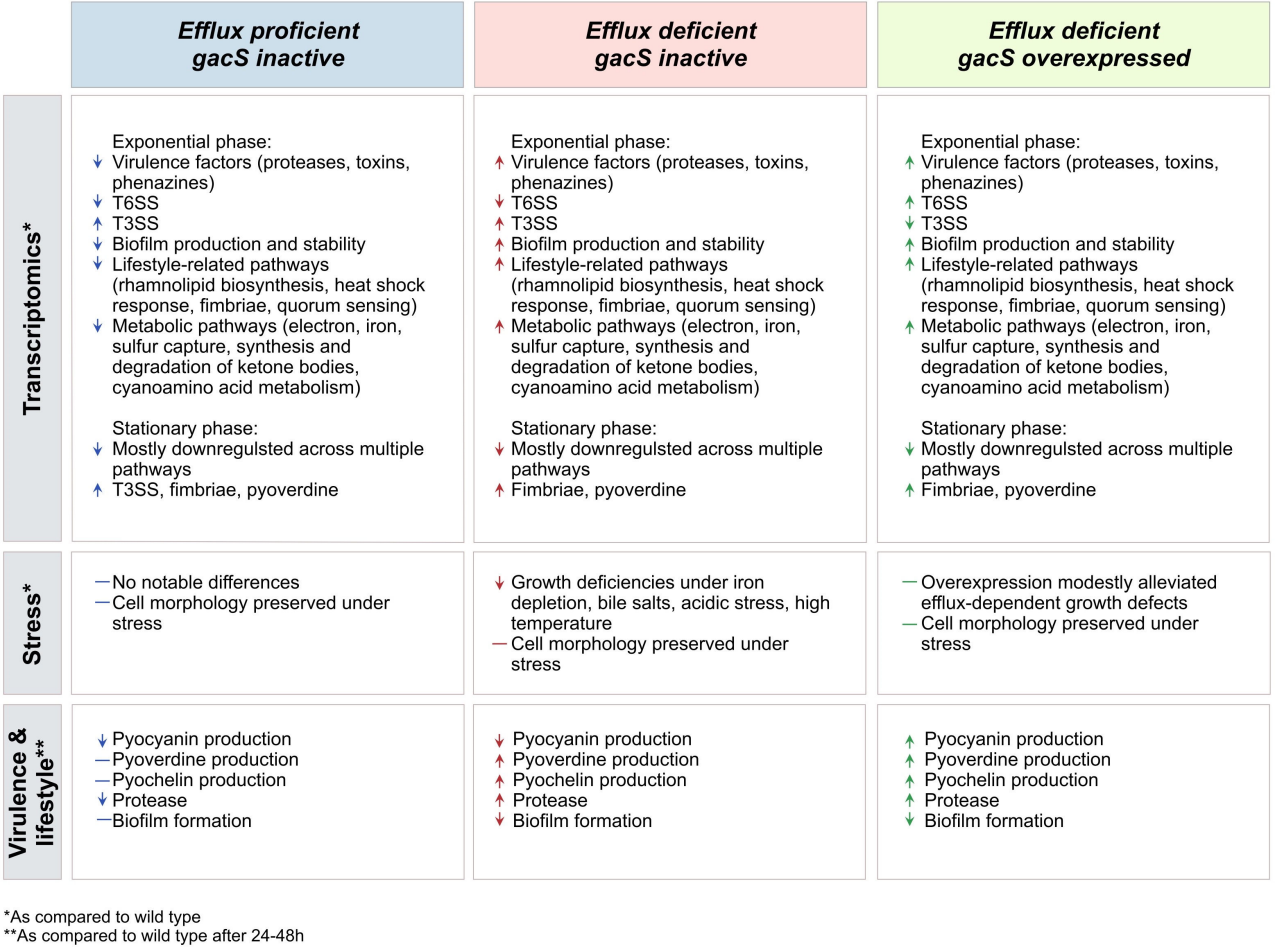
Summary of transcriptional and phenotypic changes for efflux inactivation and GacS expression.

*P. aeruginosa* virulence is a complex adaptive response to a hostile environment, which is activated by a network of two-component regulatory systems and depends on the protective activity of RND efflux pumps. We found that a major virulence regulator, GacS, is inactivated in one of the PAO1 strain lineages and analyzed a possible interplay between RND efflux function and the GacSA pathway in gene expression and associated growth phenotypes. We found that the inactivation of GacS and RND efflux pumps led to sometimes opposite effects on *P. aeruginosa* growth phenotypes and primarily affected pathways related to lifestyle, virulence, and metabolism (Fig. 6). For both mutations, the effects were more pronounced in exponentially growing cells. For both mutations, the effects on gene expression were nearly orthogonal to those induced by the transition to the stationary phase (Fig. 2). Therefore, GacS and the efflux pumps are orthogonal to each other and regulate aspects of cell physiology distinct from the cessation of growth.

The effects specific to the *gacS* and transporter mutations varied, depending on the growth phase. In exponential cells, *gacS* and Δ6 had opposite effects on cell physiology. The Δ6 genotype resulted in an increase in the expression of genes involved in virulence, lifestyle, and metabolism. Conversely, the loss of *gacS* led to downregulation in all three groups of genes (Fig. 4). In agreement, the expression of efflux pumps and GacS was also opposite to each other in growth physiology: growth defects of the RND-deficient cells under laboratory and stress conditions were aggravated by the expression of GacS (Fig. 1 and 5). In contrast, the response in the stationary phase appeared mixed and affected fewer pathways.

Several pathways, T3SS, T6SS, and macroglobulin production, displayed a similar regulation in both efflux-deficient and proficient *gacS* mutants, which was negated or reversed by the overproduction of GacS (Fig. 4). A similar pattern was observed for T3SS and T6SS in the stationary phase. Therefore, these pathways are tightly controlled by GacS. In contrast, most other pathways displayed similar expression patterns in efflux-deficient cells, especially during the exponential phase, whether functional GacS was expressed in them (Fig. 4). For these pathways, the regulation appears to be mediated by efflux, and as suggested previously, it is dictated by physiological adaptation to the lack of efflux of secondary metabolites and increased permeability of the cell envelope (23).

Nutrient deprivation is thought to be the major trigger for the activation of the GacSA regulon and virulence response (14), but signals recognized by GacS remain unknown (13). Our results suggest that these signals are unlikely to be molecules such as antibiotics, pigments, or siderophores that are recognized and expelled by RND pumps from the periplasm. Among possible candidates are ions and energetically costly sugars (13). For example, the concentration of calcium ions, which are not substrates of RND pumps, was identified as a possible switch of the GacS-dependent transition from acute to chronic virulence in *P. aeruginosa*, but this transition is mediated by a calcium-binding LadS sensor kinase positively interacting with GacS (35, 36). In addition, acid monosaccharides that are constituents of alginates in biofilm matrices and accumulate in the periplasm but are not expelled by efflux pumps were also hypothesized to be among signals recognized by GacS (13).

The phenotypic characterization of strains showed that neither of the two systems plays a major role in planktonic and sessile growth of *P. aeruginosa*. In agreement with transcriptomics, Δ6*gacS^fs^* and Δ6*gacS^fs^::LAC-gacS* strains lacking efflux functions demonstrated the deepest phenotypic defects. GacS expression alleviated at least partially the defects in the production of pyocyanin and iron acquisition, the functions associated with the activity of GacSA.

One of the limitations of these studies is the multi-level response mediated by GacSA and the complex P1116 genotype with six efflux pumps deleted. Therefore, indirect and pleiotropic interactions cannot be excluded. However, our study underscores the dominant role of RND efflux pumps in shaping the transcriptional landscape of *P. aeruginosa* and reveals the mixed contributions of RND efflux pumps and the GacS regulatory system to the bacterium’s virulence and physiology.

## MATERIALS AND METHODS

Strains and plasmids used in these studies are shown in Table 1. Unless indicated otherwise, *P. aeruginosa* strains were grown in Luria Bertani Broth (LB) (10 g tryptone, 5 g yeast extract, 5 g NaCl per liter, pH 7.0) at 37°C with shaking.

### Extraction of genomic DNA and Illumina DNA sequencing

Genomic DNA was extracted using GeneElute Bacterial Genomic DNA Kit (Sigma-Aldrich, Germany) following the manufacturer’s protocol. DNA contents were measured using NanoDrop ONE^C^ microvolume UV-Vis spectrophotometer (Thermo Scientific, USA). Genomic DNA libraries of a target size of 1,020 bp were constructed using Nextera XT DNA Library Prep and established protocols (Illumina, USA). Each library was indexed in order to multiplex for sequencing 300 bp paired-end reads using a MiSeq 600 Cycle Reagent Kit v3 on the Illumina MiSeq platform (Illumina, USA) at the Laboratory for Molecular Biology and Cytometry Research at the University of Oklahoma Health Sciences Center (OUHSC, Oklahoma City, OK, USA). A total of 68 million reads of sequencing data were collected from the run.

Mapping of the sequencing reads to the reference genome (*P. aeruginosa* PAO1, NCBI Accession no. AE004091.2) was conducted using the software CLC Genomic Workbench version 12.0.1 (formerly CLCBio; QIAGEN Aarhus, Denmark). The mapping parameters and settings were as follows: minimum length fraction 0.5 and minimum similarity 0.8. Detection of DNA variants was performed using a variant detection tool embedded in CLC Genomic Workbench with a minimum coverage of 10, a minimum count of variant reads of 2, and a minimum frequency of 100% (Table 2).

### Construction of *P. aeruginosa* knockout and complemented strains

Deletion mutants were generated from *gacS*^fs^ and Δ6*gacS*^fs^ *P. aeruginosa* through two-step allelic exchange as described by Hmelo et al. (37). Suicide vector pEXG2, carrying PCR fragments located up- and down-stream of a targeted *gacS* gene, was constructed using the Gibson Assembly technique. Overlapping flanking primers were designed to amplify the upstream and the downstream 500 bp DNA sequences of *gacS* (*gacS*-1_FOR/REV, *gacS*-2_FOR/REV; Table S2). Two fragments for the construct were then assembled into the prepared vector pEXG2 linearized by PCR (pEXG2_vector_FOR/REV; Table 2) following the Gibson Assembly reaction procedure (GeneArt Gibson Assembly HiFi Cloning Kit, Thermo Fisher Scientific, USA). Constructed pEXΔ*gacS* (Table 1) was then conjugationally transferred into recipient *P. aeruginosa* strains *gacS*^fs^ and Δ6*gacS*^fs^ to create the desired deletions (37). Homologous recombination resulted in Gm-resistant single-crossover mutants in which the plasmid was integrated site-specifically into the chromosome. Subsequently, double-crossover mutants were isolated directly using sucrose-mediated counter-selection on the no salt LB agar plates containing 15% sucrose (37). Deletions were finally confirmed by PCR.

Chromosomal insertion of the *gacS* gene was performed as described by Choi and Schweizer (20). To amplify the gene encoding the GacS sensor involved in differentiation and virulence programs of *P. aeruginosa*, a set of primer pairs was designed (*gacS*_FOR/*gacS*_REV) with the following restriction sites BamHI and HindIII (Table S2). The *gacS* DNA fragment was amplified by PCR using the genomic DNA of *P. aeruginosa* PAO1 as a template. The corresponding product was cloned into pUC18T-mini-Tn7T-LAC vector to generate pUC18T-mini-Tn7T-LAC-*gacS* expressing sensor/response regulator hybrid under the control of the IPTG-inducible promoter (Table 1). Triparental mating was used to construct *gacS*^fs^ and Δ6*gacS*^fs^ cells overproducing *gacS*. The mini-Tn7 insertion was verified using PCR with glmS-specific primers (Pa glmS Up and Pa glm S Down, as well as Pa glm S Down and Pa Tn7R) and by sequencing (Table S2). mRNA extraction and sequencing

All steps, including mRNA extraction, library preparation, and sequencing, were performed as previously described (23). Briefly, high-quality total RNA was harvested from *gacS*^+^, *gacS*^fs^, Δ6*gacS*^fs^, and Δ6*gacS*^fs^::LAC-*gacS* mutant cells (Table 1) grown in LB broth until mid-exponential (OD600 ~0.5) and stationary phase (OD600 ~2) in two biological replicates using the hot phenol method. The removal of ribosomal RNA was performed using the Ribo-Zero Bacteria Kit (Illumina, USA), and cDNA libraries were generated with the TruSeq RNA LT v2 kit (Illumina, USA) according to established protocols provided by the manufacturer. Samples were normalized, and all libraries were pooled onto a 2 × 150 bp paired-end sequencing run on the Illumina MiSeq. A total of 48 million reads (7.5 Gb) of sequencing data were collected from the run.

### Transcriptomic analysis

Read counts of every gene were normalized to the median logarithmic expression level of each sample; 113 genes had an average expression count of less than one in all samples and were excluded from further analysis. We also excluded 18 genes associated with known genetic variations between the strains, including *gacS* and efflux transporters. The normalized read counts of the remaining 5,476 genes were modeled as a negative binomial distribution and compared to *gacS^+^* to evaluate the significance of expression changes, as previously described (38). We found, respectively, 2,107 and 1,293 genes, whose expression was significantly different (fold change >2, false discovery rate, FDR, <0.05) from the reference in at least one of the variants in the exponential or stationary phase. The replica-averaged expression levels in each strain were normalized to those in the reference *gacS^+^* strain, and cluster analysis was performed on the logarithms of the resulting ratios. The elbow method was used to determine the optimal number of clusters. Clusters with fewer than 10 genes were merged with their nearest neighbor. For both exponential and stationary cells, the largest clusters were comprised of the least affected genes and included most of the genes that were judged insignificantly affected according to the initial fold-change/FDR analysis, + out of 3,369 for exponential and 3,911 out of 4,136. All genes in these clusters were judged insignificantly affected. Overall, we identified 842 significantly affected genes in the exponential samples that were grouped into 13 clusters, whereas the exponential samples grouped 589 genes in 11 significant clusters.

Pathway enrichment analysis was performed on each cluster, utilizing information pooled from multiple sources, including the Kyoto Encyclopedia of Genes and Genomes (KEGG), Virulence Factor Database (VFDB), Pseudomonas Genome Database (PseudoCAP), psedudomonas.com database, and additional sources listed by Zhao et al. (39). Significantly enriched pathways in each cluster were determined by modeling them as hypergeometric distributions and adjusting their *p*-values using the Benjamini-Hochberg method (38, 39). Enriched pathways with fewer than two genes were excluded from further analysis.

### Growth under infection-relevant stress conditions

In order to investigate the stress response of *P. aeruginosa* and related mutants (Table 4), we analyzed their growth curves under host-related stress conditions as reported by Avican et al (28) with modifications. Overnight cultures were diluted to an OD_600_ of 0.001 and inoculated at 5 × 10^3^ cells into a 96-well plate containing LB broth and exposed to five stresses, separately (Table 4). Optical densities were monitored in a Tecan Spark 10M plate reader every 30 min at 37°C or 41°C when indicated. As expression of *gacS* in the Δ6*gacS*^fs^::LAC-*gacS* strain was under an inducible promoter (Table 1), cells were induced with 0.1 mM isopropyl β-D-1 thiogalactopyranoside (IPTG) if necessary. No effect of IPTG was found on the growth of *P. aeruginosa* strains under these conditions (Fig. S5) (34). Experiments were performed in at least two replicates, and data were collected from two independent experiments. Area under the growth curve (AUC) (growth potential) was used as an additional growth metric, which correlated with both the growth rate and the cell density (40). Multivariate statistical techniques (principal component analysis, PCA) were applied to assess the association between strains growing under stress conditions.

### Minimal inhibitory concentration (MIC)

MIC was determined using the 2-fold broth dilution method as described previously (41). Briefly, cells were grown to OD_600_ = 1.0 and inoculated into a 96-well plate at a concentration of 5 × 10^4^ CFU/mL. Plates were set up with a 2-fold increasing concentration of antibiotics in 100 µL of LB broth per well. Plates were incubated at 37°C with shaking for 16–20 h. When needed, the expression of the *gacS* gene was induced by the addition of 0.1 mM IPTG.

### Pyocyanin extraction and quantification

Extraction and quantification of pyocyanin was performed as described by Lee et al. (42). Constructed *gacS* mutants were grown overnight in LB broth at 37°C with shaking; 3 mL of chloroform was then added to 5 mL culture and shaken vigorously for 30 min at room temperature. The solvent phase was collected and mixed with 1 mL of 0.2 N HCl and shaken for an additional 30 min. The quantity of pyocyanin was measured at 520 nm and normalized to cell density registered at 600 nm.

### Biofilm formation

Biofilm formation was tested using the Calgary Biofilm Device following the manufacturer’s protocol with some modifications (43). Microbial cells were seeded into a 96-well plate (10^5^ CFU/mL), and a 96-peg lid was placed on the plate to allow cells to grow for 48 h. Biofilm was formed on the pegs attached to the lid. Pegs were transferred to a fresh plate, washed in a sterile PBS buffer, and stained with 0.1% crystal violet for 30 min. Stained pegs were washed again in PBS buffer before extraction in ethanol for 15 min in a fresh plate. Optical density measurements were carried out in a microplate reader at 590 nm.

### Siderophore production

Pyoverdine was quantified as previously described (24, 44). Briefly, the cells were grown overnight in LB with shaking at 37°C. They were diluted 1:100 in fresh LB media and grown for the indicated time at 37°C. The supernatant was diluted in 100 mM Tris-HCl (pH 8.0), and pyoverdine was determined by measuring the absorbance at 405 nm. The values were normalized to OD_600_.

Pyochelin measurements were carried out as previously described (45). Cultures were grown for the indicated time points, and the cell-free supernatant was collected. Pyochelin was extracted with ethyl acetate from acidified supernatants. The top organic ethyl acetate layer was concentrated using a SpeedVac and quantified at OD_313_ in a 1:1 mixture of methanol: water. The values were normalized to OD_600_.

### Protease activity

Protease secretion was determined using the azocasein assay as mentioned elsewhere (24, 46). Briefly, the cells were grown for 24 h with shaking at 37°C. 350 µL of reaction buffer containing 1% azocasein, 0.5% NaHCO_3_, and 0.1M Tris-HCl (pH 8.0) was added to 150 µl of culture supernatant and incubated at 37°C with shaking for 20 min; 1 mL of 7% perchloric acid was added, followed by brief centrifugation. Clear supernatant was added to 150 µL of 10M NaOH, and OD values at 430 nm were measured.

## Data Availability

All data generated during this study are included in the article. Sequencing results have been uploaded to the GEO database under accession number GSE284138. Strains and plasmids are available upon request.

## SUPPLEMENTAL MATERIAL

Supplemental material aem.01223-25-s0001.pdf Tables S1 and S2; Fig. S1 to S5.

ASM does not own the copyrights to Supplemental Material that may be linked to, or accessed through, an article. The authors have granted ASM a non-exclusive, world-wide license to publish the Supplemental Material files. Please contact the corresponding author directly for reuse.
