# Supplementary material for: Functional interplay between RND efflux pumps and GacS in Pseudomonas aeruginosa
Source: Appl Environ Microbiol. 2025 Aug 18;91(9):e01223-25. doi: 10.1128/aem.01223-25 (PMC12442375; doi:10.1128/aem.01223-25)

Supplementary Information for:

Functional interplay between Resistance-Nodulation-Division efflux pumps and GacS in *Pseudomonas aeruginosa*

Justyna Adamiak, Charles Bergen, Laiba Ajmal, Sidhvi Reddy, Paolla Gruber Anderson, Valentin V. Rybenkov* and Helen I. Zgurskaya*

University of Oklahoma, Department of Chemistry and Biochemistry, Norman, OK 73019, USA

* Corresponding authors: elenaz@ou.edu and valya@ou.edu

Content:

Table S1. Fold changes (log2) in the expression of extracellular sigma factors and selected representatives of their regulons.

Table S2. List of primers used in this study.

Figure S1. Levels of *gacS* transcripts in exponential and stationary cells of the indicated *P. aeruginosa* cells as determined by RNA-seq experiments.

Figure S2. Growth curves of *gacS* mutants under different stresses.

Figure S3. Cell morphology of the studied *P. aeruginosa* strains under normal and stress conditions.

Figure S4. Venn diagrams illustrating *P. aeruginosa* gene expression altered by mutations in *gacS* and RND efflux pumps.

Figure S5. Growth curves of Δ6gacS^{fs} and the complemented strain in the presence and absence of 0.1 mM IPTG.

Table S1. Fold changes in the expression of extracellular sigma factors and selected genes under their regulation.

**Gene**	Log2 fold change exponential phase			Log2 fold change stationary phase		
	***gacS*^{fs}**	**Δ6*gacS*^{fs}**	**Δ6*gacS*^{fs}:: LAC-*gacS***	***gacS*^{fs}**	**Δ6*gacS*^{fs}**	**Δ6*gacS*^{fs}:: LAC-*gacS***
algU	1.24	-1.08	-1.60	0.11	-0.09	-0.29
SigX	0.59	-0.13	-0.37	-0.06	-0.54	-0.15
sbrI PA2896	-1.10	-1.64	-0.99	-0.23	-0.51	0.31

Table S2. List of primers used in this study.

**Primer name**	**5'-3' Sequence**
pEXG2_vector_FOR	TATCTGCCGAACTCTAGAGGATCCCCGGGCTCGAG
pEXG2_vector_REV	TCGGCTGCGACGACCTGCAGAAGCTTGC
gacS-1_FOR	CTGCAGGTCGTCGCAGCCGAAGCCGG
gacS-1_REV	CTCGGCATCAGCCTCGACTCCAGCGAAC
gacS-2_FOR	GAGTCGAGGCTGATGCCGAGATCCTTGAACACAC
gacS-2_REV	CCTCTAGAGTTCGGCAGATAATCGTCGGCG
*gacS*_FOR	AATTGGATCCGTGTTCAAGGATCTCGGCATCAAGG
*gacS*_REV	ATATAAGCTTTCAGAGTTCGCTGGAGTCGAGG
Pa glmS Up	CTGTGCGACTGCTGGAGCTGA
Pa glms Down	GCACATCGGCGACGTGCTCTC
Pa Tn7R	CACAGCATAACTGGACTGATTTC

Figure S1. Levels of *gacS* transcripts in exponential and stationary cells of the indicated *P. aeruginosa* cells as determined by RNA-seq experiments. Reads Per Kilobase per Million reads mapped (RPKMM) are shown as averages of two independent biological repeats with experimental errors.

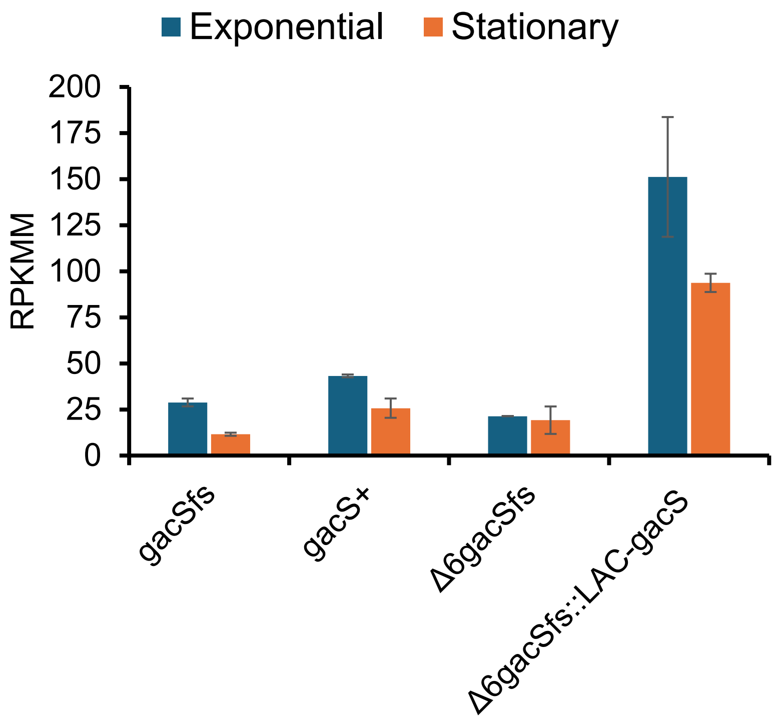

Figure S2. Growth curves of *gacS* mutants under different stresses. A. LB broth, 37ºC. **B-C.** Limited iron conditions in the presence of 125 µM and 250 µM iron chelator 2,2'-dipyridyl, respectively. **D-E.** Bile salt stress in the presence of 0.125% and 0.5% sodium deoxycholate. **F.** Osmotic stress in the presence of 0.5 M NaCl. **G.** Acidic stress in LB broth pH=4.6. **H.** High temperature stress in LB broth 41°C.

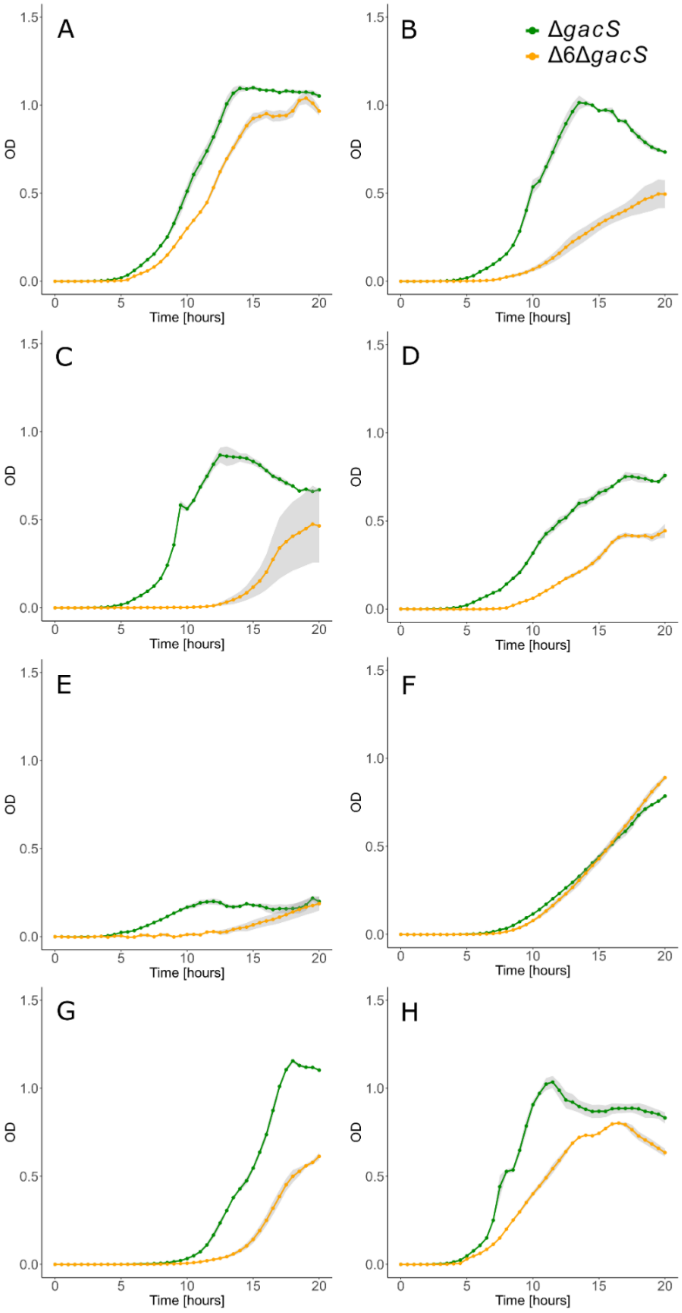

Figure S3. Phase-contrast microscopy of *P. aeruginosa* strains under normal and stress conditions. (A) *gacS*$^{+}$, (B) *gacS*^{fs}*, (C) ΔgacS*, (D) *gacS*^{fs}*::LAC-gacS,* (E) *Δ6gacS*^{fs}*,* (F) *Δ6ΔgacS,* (G) *Δ6gacS*^{fs}*::LAC-gacS.*

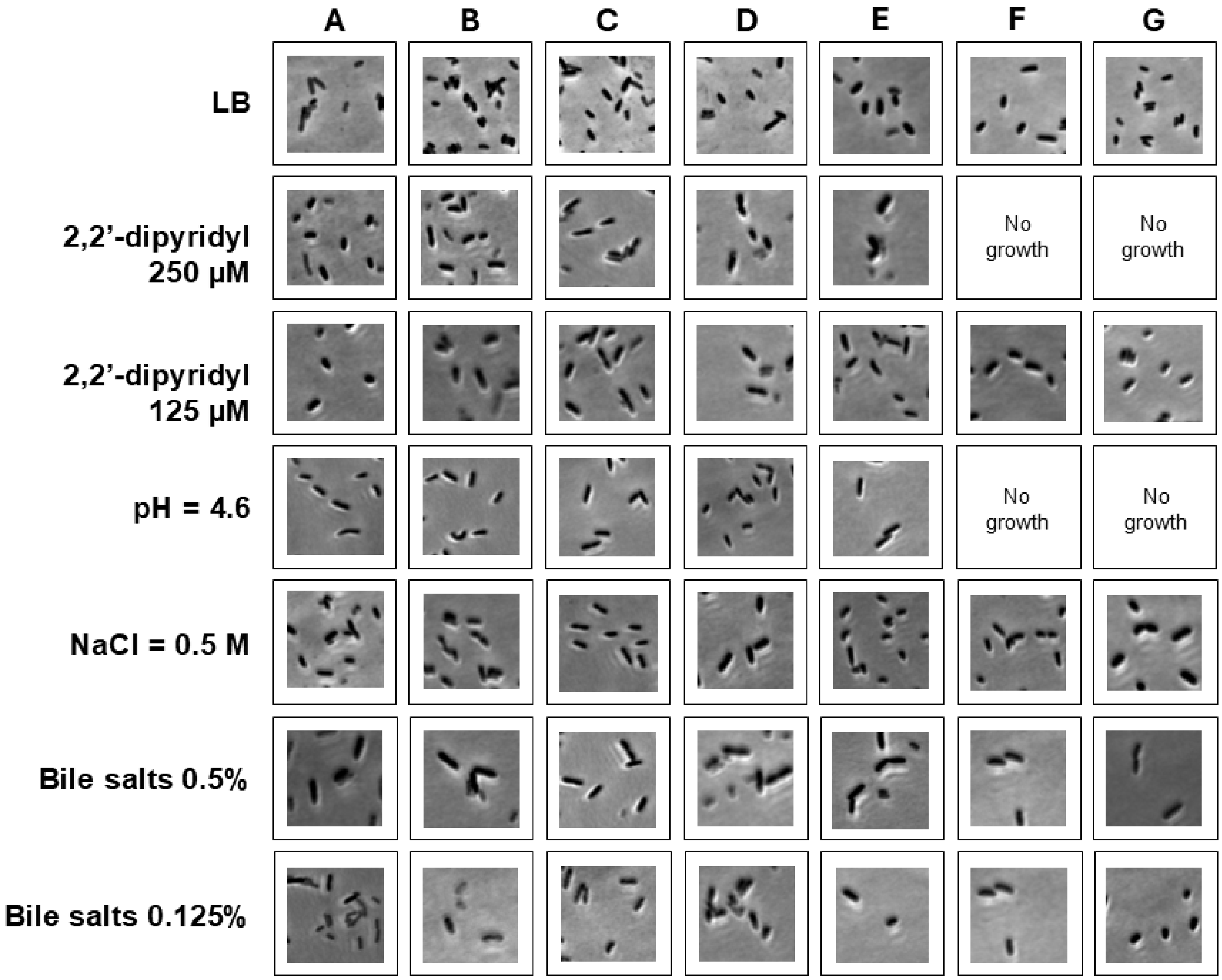

Figure S4. Venn diagrams illustrating *P. aeruginosa* gene expression altered by mutations in *gacS* and RND efflux pumps.

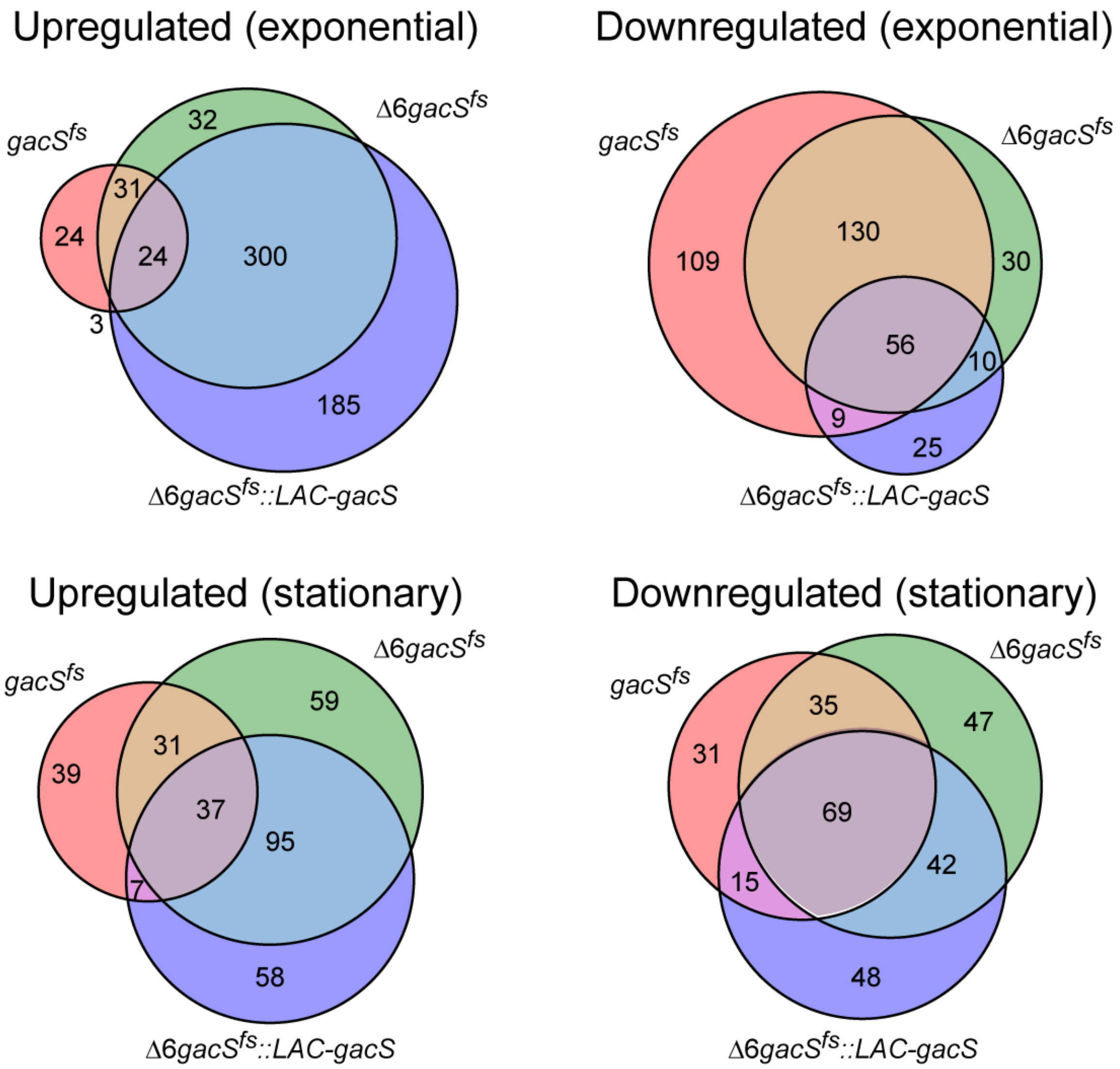

Figure S5. Growth curves of Δ6gacS^{fs} and the complemented strain in the presence and absence of 0.1 mM IPTG.

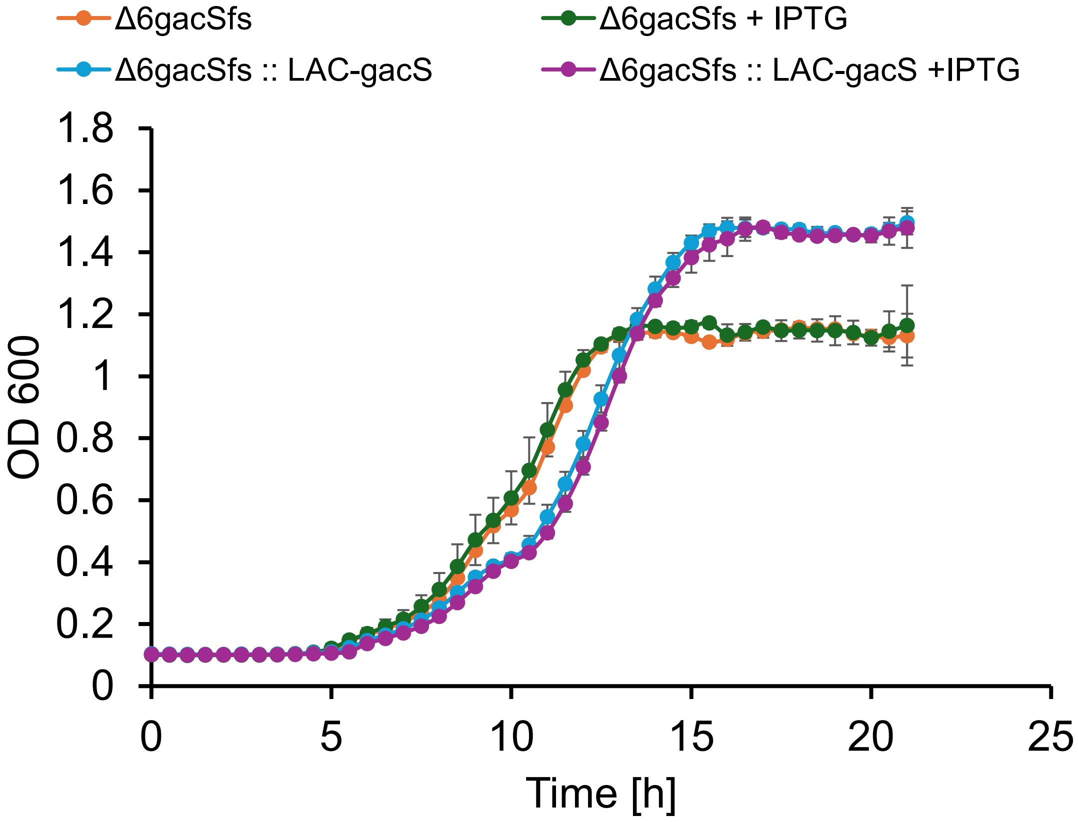

Supplement: Supplemental material — Tables S1 and S2; Fig. S1 to S5. [file aem.01223-25-s0001.pdf]
